# The Profile of Phenolic Compounds Identified in Pitaya Fruits, Health Effects, and Food Applications: An Integrative Review

**DOI:** 10.3390/plants13213020

**Published:** 2024-10-28

**Authors:** Vinicius Serafim Coelho, Daniela Gomes de Moura, Lara Louzada Aguiar, Lucas Victor Ribeiro, Viviane Dias Medeiros Silva, Vinícius Tadeu da Veiga Correia, Angelita Cristine Melo, Mauro Ramalho Silva, Ana Cardoso Clemente Filha Ferreira de Paula, Raquel Linhares Bello de Araújo, Julio Onesio Ferreira Melo

**Affiliations:** 1 Departamento de Alimentos, Faculdade de Farmácia, Campus Belo Horizonte, Universidade Federal de Minas Gerais, Belo Horizonte 31270-901, MG, Brazil; viniciusserafimcoelho16@gmail.com (V.S.C.); danielagomes2014@outlook.com (D.G.d.M.); laralaguiar.doc@gmail.com (L.L.A.); viniciustvcorreia@gmail.com (V.T.d.V.C.); raquellba.linhares@gmail.com (R.L.B.d.A.); 2 Departamento de Ciências Exatas e Biológicas, Campus Sete Lagoas, Universidade Federal de São João del-Rei, Sete Lagoas 36307-352, MG, Brazil; luc4sribeiro13@gmail.com (L.V.R.); vivianedms05@gmail.com (V.D.M.S.); 3 Curso de Farmácia, Campus Centro-Oeste, Universidade Federal de São João del-Rei, Divinópolis 35501-296, MG, Brazil; angelitamelo@ufsj.edu.br; 4 Departamento de Bioquímica e Imunologia, Campus Pampulha, Universidade Federal de Minas Gerais, Belo Horizonte 31270-901, MG, Brazil; mauroramalhosilva@yahoo.com.br; 5 Departamento de Ciências Agrárias, Campus Bambuí, Instituto Federal de Educação, Ciência e Tecnologia de Minas Gerais, Bambuí 38900-000, MG, Brazil; ana.paula@ifmg.edu.br

**Keywords:** dragon fruit cactus, *Hylocereus*, phytochemicals, polyphenols

## Abstract

Objective: This integrative review aimed to identify the phenolic compounds present in pitayas (dragon fruit). Methods: We employed a comprehensive search strategy, encompassing full-text articles published between 2013 and 2023 in Portuguese, English, and Spanish from databases indexed in ScienceDirect, Capes Periodics, Scielo, and PubMed. The study’s selection was guided by the question, “What are the main phenolic compounds found in pitaya fruits?”. Results: After screening 601 papers, 57 met the inclusion criteria. The identified phytochemicals have been associated with a range of health benefits, including antioxidant, anti-inflammatory, and anxiolytic properties. Additionally, they exhibit promising applications in the management of cancer, diabetes, and obesity. These 57 studies encompassed various genera, including *Hylocereus*, *Selenicereus*, and *Stenocereus*. Notably, *Hylocereus undatus* and *Hylocereus polyrhizus* emerged as the most extensively characterized species regarding polyphenol content. Analysis revealed that flavonoids, particularly kaempferol and rutin, were the predominant phenolic class within the pulp and peel of these fruits. Additionally, hydroxycinnamic and benzoic acid derivatives, especially chlorogenic acid, caffeic, protocatechuic, synaptic, and ellagic acid, were frequently reported. Furthermore, betalains, specifically betacyanins, were identified, contributing to the characteristic purplish-red color of the pitaya peel and pulp. These betalains hold significant potential as natural colorants in the food industry. Conclusion: Therefore, the different pitayas have promising sources for the extraction of pigments for incorporation in the food industry. We recommend further studies investigate their potential as nutraceuticals.

## 1. Introduction

*Cactacea* thrive in arid and adverse conditions in their natural habitats, which include intense heat, drought, low soil nutrient availability, and constant herbivore attack [1]. In response, these cacti synthesize a diverse array of bioactive secondary metabolites, such as phenolic compounds, which constitute their chemical defense system and facilitate their adaptation to these unfavorable conditions [1,2]. *Cactaceae* is a large plant family, encompassing 1.922 species distributed across 130 genera [3]. Notably, a significant number of these species produce edible fruits with distinct sensory characteristics. These fruits typically possess sweet, slightly acidic pulp and exhibit a diverse array of appealing colors, ranging from yellow to purple-red, as exemplified by pitaya fruits [3,4]. The terms pitaya, pitahaya, or dragon fruit are general names for a diverse group of genera in the family *Cactaceae* [5].

The genus *Hylocereus* contains several commercially important pitayas species [5]. In the most recent review, the most commercially important species of pitayas were included in the genus *Selenicereus*: *S. undatus*, *S. costaricenses*, *S. setaceus*, and *S. megalanthus* [6].

**Figure 1:**
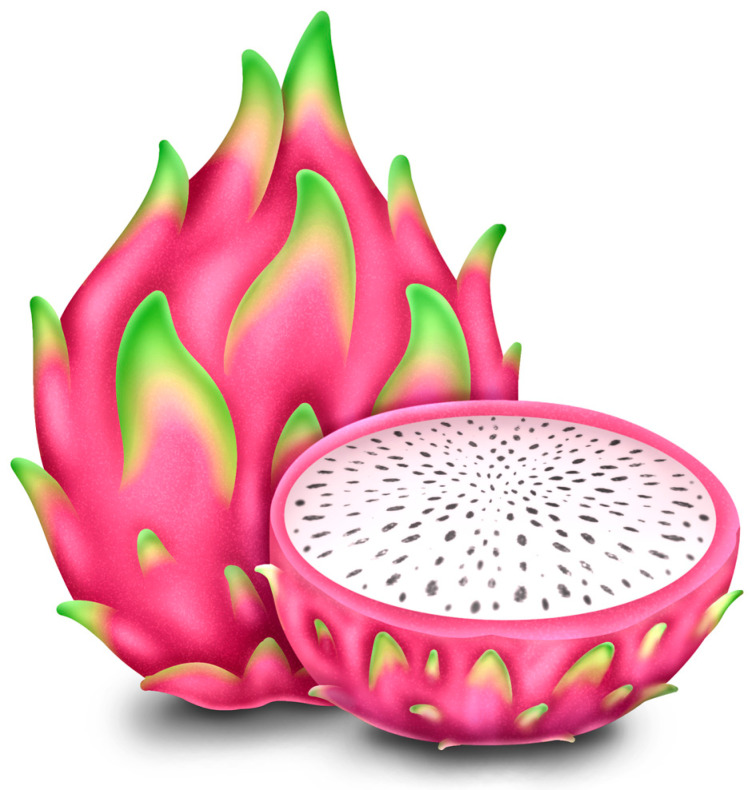
*Selenicereus undatus*. Illustration made by Ribeiro, L.V. (2024).

*Selenicereus undatus* (Figure 1), the most well-known and widely cultivated pitaya, is prized for its white, sweet pulp with edible black seeds and vibrant pink peel [7]. This drought-tolerating species is native to tropical and subtropical regions of the Americas and is distinguished by its fruits with remarkable antioxidant potential due to the high content of phenolic compounds and betalains [8]. On average, *Selenicereus undatus* fruit reaches 17 cm in diameter, with the peel comprising approximately 30 to 45% of the total weight and the remaining portion consisting of the edible pulp [5,7,8,9].

**Figure 2:**
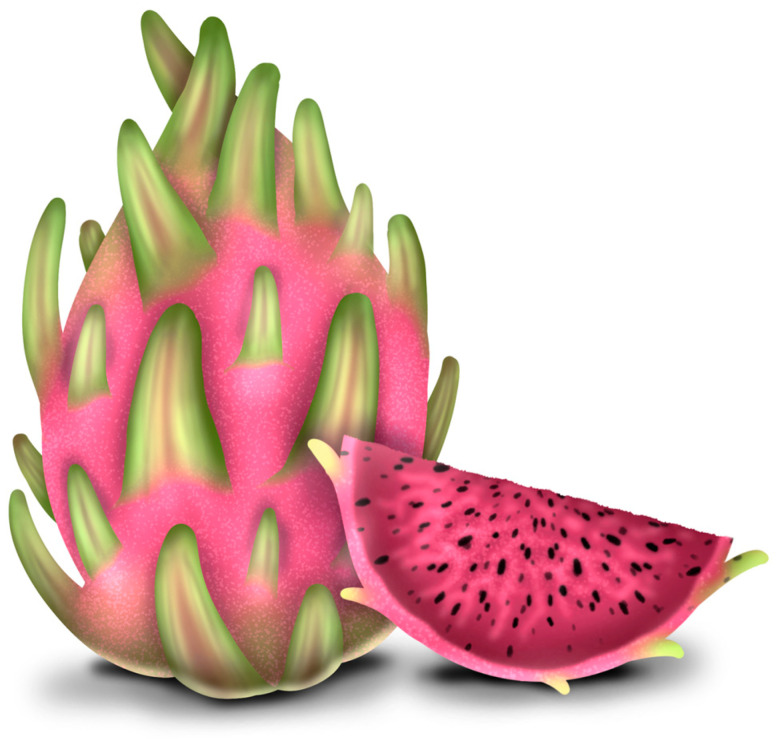
*Hylocereus polyrhizus*. Illustration made by Ribeiro, L.V. (2024).

*Hylocereus polyrhizus* (Figure 2) is another commonly traded species of the genus *Hylocereus* and is native to Latin America. However, the flesh is pinkish-red in color, with numerous black seeds and bright red peel [10]. This fruit weighs about 300 to 500 g, with the peel being about 33% of the total fruit weight [11]. The fruit’s peel and pulp stand out for the presence of bioactives, polyphenols such as phenylpropanoids, simple phenols, flavonoids, and tannins [7,10,11,12,13].

**Figure 3:**
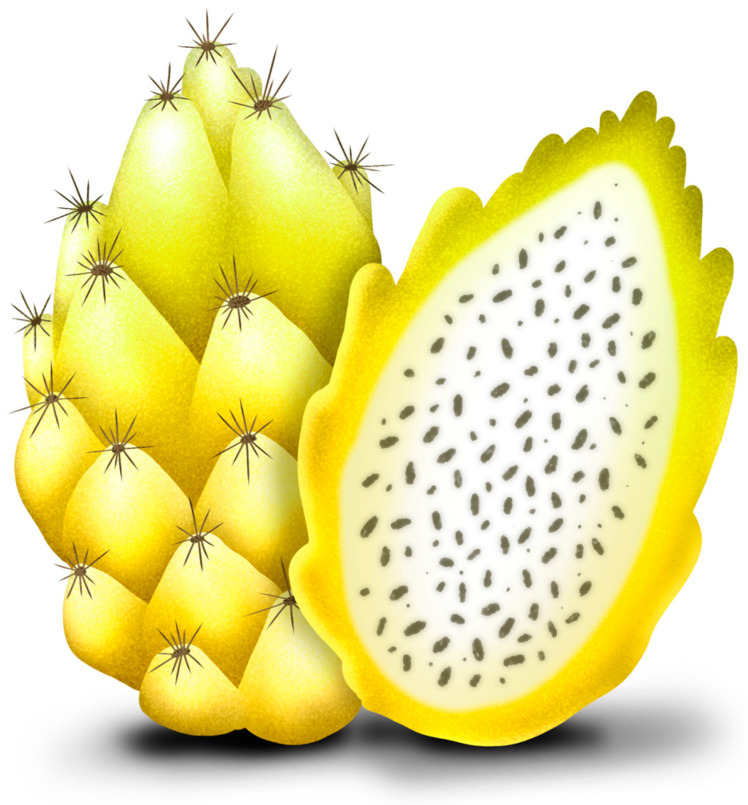
*Selenicereus megalanthus*. Illustration made by Ribeiro, L.V. (2024).

*Selenicereus megalanthus* (Figure 3), commonly known as yellow pitaya, originates from cacti cultivated in the Caribbean region of South America, particularly Colombia [10]. This species is distinguished by its yellow peel, a white pulp with black seeds found within. The peel typically comprises 20 to 40% of the total fruit weight [14]. Similar to the other fruits of this genus, it is a source of antioxidant compounds. However, further investigations are necessary to fully elucidate its phytochemical potential [14,15,16,17].

**Figure 4:**
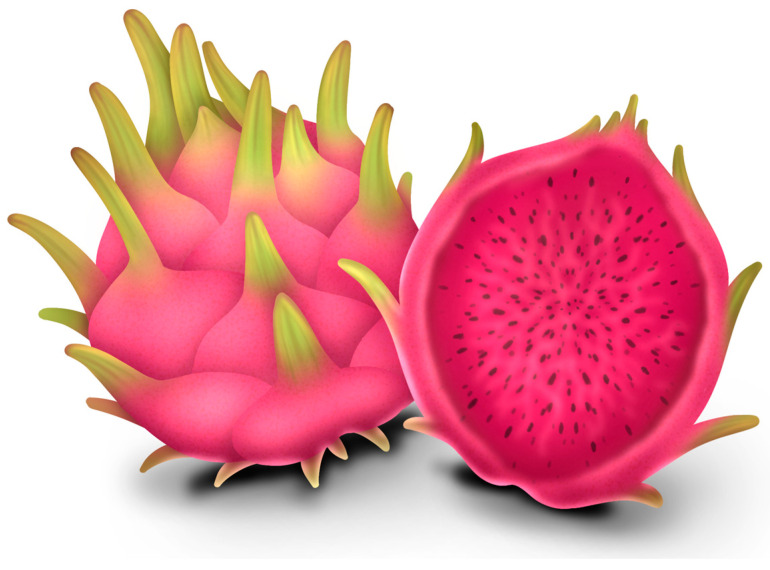
*Selenicereus costaricensis*. Illustration made by Ribeiro, L.V. (2024).

*Selenicereus costaricensis* (Figure 4), a Latin America’s native pitaya, resembles *Hylocereus polyrhizus*, with red pulp with reddish pink peel [11]. The pulp corresponds to approximately 67% [18]. In addition, similar to other species, they have compounds with beneficial effects on health, emphasizing their functional potential. In particular, it stands out for its high content of betalains [16,18,19].

**Figure 5:**
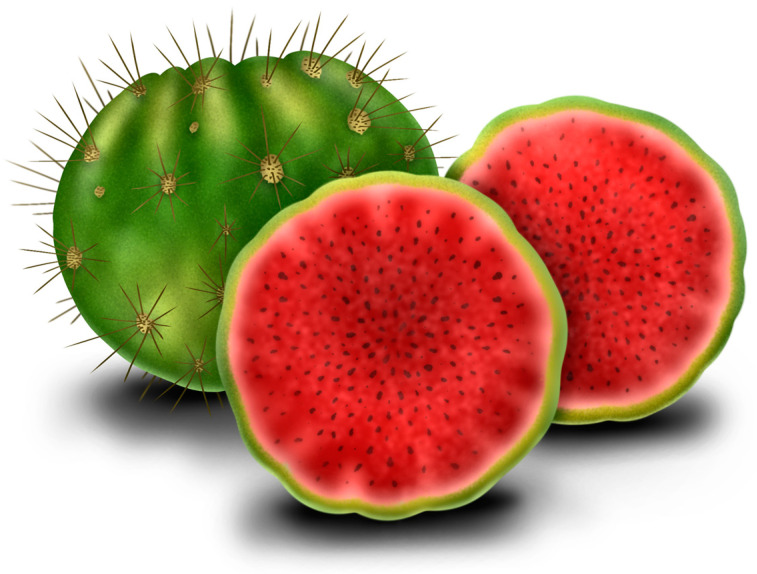
*Stenocereus thurberi*. Illustration made by Ribeiro, L.V. (2024).

*Stenocereus thurberi* (Figure 5), a cactus fruit native to the arid and semi-arid regions of northwestern Mexico, stands out as a relatively understudied and explored species compared to its congeners [20]. These pitayas produce spherical or oval fruits with a green, thorn-covered peel and orange-red pulp [21]. Notably, the fruit holds a promise due to its content of betalains, phenolic compounds, and its use as a natural dye source [20,22].

**Figure 6:**
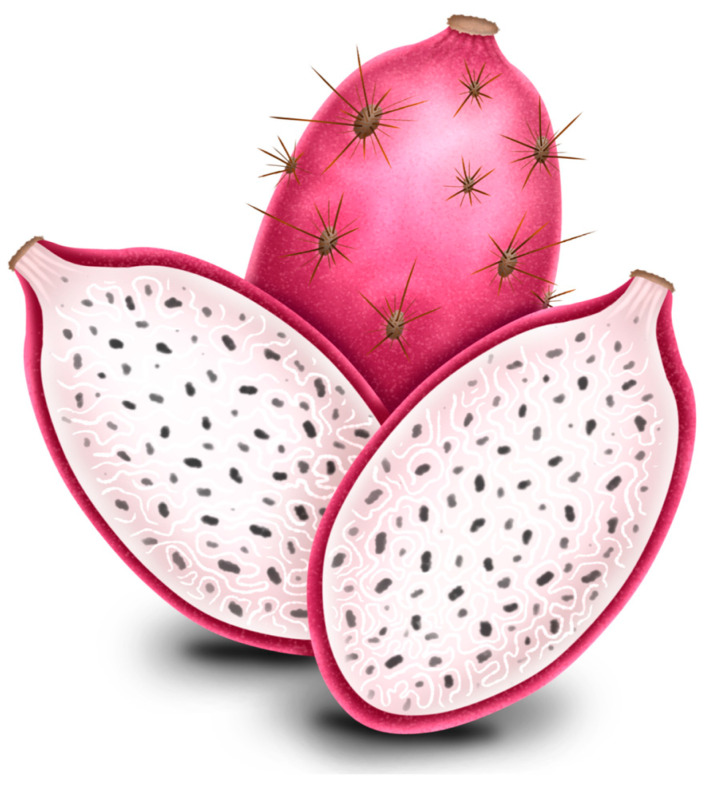
Saborosa do Cerrado (*Selenicereus setaceus* Rizz). Illustration made by Ribeiro, L.V. (2024).

Brazil’s Cerrado and Caatinga biomes harbor native pitayas species belonging to the genera *Selenicereus* [23,24]. One such exemple is the *Saborosa do Cerrado*, “Cerrado’s Tasty” (*Selenicereus setaceus* Rizz.) (Figure 6), also known as pitaya-do-cerrado, pitaya-vermelha, minipitaya, or baby pitaya [25]. These species thrive naturally in rocky areas, sandy soils, and on tree trunks. The fruit boasts white, juicy pulp with edible black seeds, enveloped by a reddish peel with purple hues. Its shape resembles the yellow pitaya (*Selenicereus megalanthus*); however, it is smaller (30 to 80 g) and has a higher pulp-to-peel ratio (approximately 75%) [25]. The fruit’s exotic characteristics—color, flavor, and aroma—capture the consumer’s interest. However, research on its nutritional value and bioactive compounds remains limited [23,24,25].

The vibrant hues of pitaya fruits, ranging from deep purple to vibrant yellow, stem from the presence of betalains, a unique class of water-soluble nitrogen-containing pigments [26]. These pigments are synthesized from betalamic acid, a tyrosine derivative that forms the structural backbone of all betalains. Betalains are further categorized into betacyanins, which impart red-purple shades, and betaxanthins, which lend yellow-orange to fruits [22,26,27]. Despite their widespread occurrence in plants, betalains are found in relatively few edible plant matrices. The most well-known sources include beetroot, prickly pear, amaranth, and pitaya, all of which belong to the order Caryophyllales [26]. These pigments, along with phenolic compounds present in the peel and pulp of pitaya fruits, impart potent antioxidant properties and are associated with beneficial health effects [6,19].

Phenolic compounds are present in pitaya pulp, seed, and peel, mainly comprising the class of flavonoids and a variety of phenylpropanoids [7,9,22,28,29]. These compounds have been linked to a range of potential health benefits. These include antioxidant and anti-inflammatory activities, improved lipid profiles, regulation of blood sugar levels, and even the potential to contribute to the treatment of Alzheimer’s disease [26,27,28,29,30].

The growing interest in characterizing the phytochemical profile in pitaya fruits stems from two key aspects: the bioactive potential for health (due to their proven bioactivity) and the functional food ingredients. Furthermore, the vibrant betalain pigments, particularly abundant in the peel and colored pulp (excluding white-fleshed varieties), present a potential alternative to synthetic dyes in the food industry [18].

The pulp of the fruit is the part usually consumed; however, the peel can also be characterized as a potential source of health-promoting polyphenols. The by-product of pitaya processing is mainly composed of the peel, accounting for about 30% to 45% of the whole fruit. This bioresidue is a source of natural pigments, such as betalains, as well as polysaccharide dietary fibers and, mainly, phenolic compounds [9].

Understanding the chemical and functional potential that different species of pitayas can present and the limitation of other review studies on phytochemicals found in different species of cactus fruits and their different fractions, the objective of this work is to describe the main phenolic compounds found in pitayas, establishing the profile of the main phytochemicals observed, whether in the pulp, peel, or seeds of these fruits, in addition to the health effects associated with the presence of bioactives and the development of food matrices with added functional value, attributed to the incorporation of these phytochemicals. Thus, in order to achieve this objective, the following guiding question was used: “What are the main phenolic compounds found in pitaya fruits?”.

## 2. Results and Discussion

**Figure 7:**
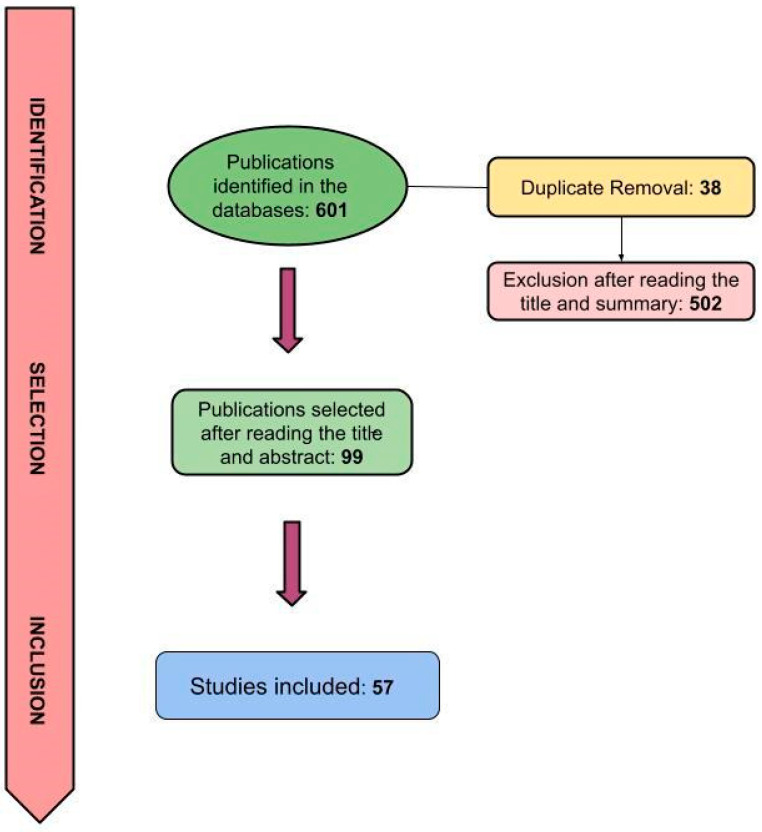
Flowchart for the selection of studies by stages.

The guiding question led to the selection of 601 articles. After reading the title and abstract, 99 articles were included, followed by the removal of 38 duplicates. After reading the articles in full, only 57 were included in the study and used for the interpretation and discussion of the results (Figure 7).

The species found and characterized for the presence of phenolic compounds and betalains of the 57 studies included in the review were *Hylocereus undatus* (27), *Hylocereus polyrhizus* (27), *Hylocereus costaricensis* (5), *Hylocereus megalanthus* (4), *Hylocereus* spp. (4), *Hylocereus lameri* (1), *Stenocereus thurberi* (1), *Stenocereus huastecorum* (1), *Stenocereus pruinosus* (1), *Stenocereus stellatus* (1), *Selenicereus setaceus* (2), and *Selenicereus megalanthus* (1). Some studies characterized more than one species. The most characterized genus in terms of the presence of polyphenols is *Hylocereus*, representing 91% of the studies, followed by *Stenocereus* (4%) and *Selenicereus* (3%).

### 2.1. Phenolic Compounds Profile

Pitaya fruits boast a diverse array of flavonoids and phenolic chemical compounds, present not only in the edible pulp and seed but also in the peel (Table 1). These phenolic compounds are particularly noteworthy for their potential health benefits, mainly attributed to the antioxidant properties [22]. These are classified as antioxidants due to the fact that they have redox properties, allowing them to act as reducing agents, oxygen inhibitors, hydrogen donors, and metal chelators [20].

The chemical and nutritional characteristics of pitayas can vary depending on their origin [12]. Thus, a phytochemical survey of different genera and species of pitayas may present distinct phenolic profiles [16]. In pitayas of the genus *Stenocereus*, such as *Stenocereus pruinosus*, *Stenocereus stellatus*, and *Stenocereus thurberi*, flavonoids such as quercetin, kaempferol, rutin, and catechin have been reported in the pulp and peel [20,22,30]. Phenylpropanoids, such as hydroxycinnamic and hydrobenzoic acids and derivatives, have also been detected in this genus, including ferulic acid, caffeic acid, p-coumaric acid, and gallic acid [20,22,30]

The pulp of *Hylocereus undatus* (white pulp) and *Hylocereus polyrhizus* (purple pulp) fruits without the presence of seeds were also characterized for the profile of phenolic compounds [6]. The fruits were cultivated under the same edaphoclimatic conditions and characterized mainly in terms of bioactive profile and antioxidant capacity. In total, 20 phenolic compounds were found in *H. polyrhizus* and 17 in *H. undatus*. Notably, ferulic acid was exclusively detected in the purple-fleshed fruit. Additionally, *H. polyrhizus* exhibited a higher content of rutin and hesperidin—flavonoids recognized for their potent antioxidant and anti-inflammatory properties [7,31]. Conversely, chlorogenic acid, a hydroxycinnamic acid, was the most reported phenolic compound in *H. undatus*. This substance is reported especially in coffee and tea and linked to antioxidant, anti-inflammatory, lipid metabolism, and glucose regulation activity [29,32,33].

While typically discarded during consumption, the peel of *Hylocereus polyrhizus* and *Hylocereus undatus* fruits holds immense potential as a source bioactive of phenolic compounds associated with health benefits and food applications. A study by Tang et al. [12] explored the phenolic profile of these peels using different extraction methods (acid hydrolysis, basic hydrolysis, and compound enzymes), and normally, the quantification of these phytochemicals is associated only with free phenolic compounds, facially extractable from the matrix. In addition to the common extraction, using the solvent methanol, the authors also applied acid and basic extraction and the incorporation of compound enzymes (cellulase, hemicellulose, and pectinase) in order to also extract the phenolics bound to the matrix, and in this study, the bound compounds represented 50% of the phytochemicals found in the peel of the two species studied [7].

The study identified 37 individual phenolic compounds, distributed in the chemical classes of benzoic acid derivatives and derivatives, hydroxycinnamic acids and derivatives, and flavonoids. Caffeic, ferulic acid, and *p*-coumaric acid were predominantly the bound phenolics, whereas chlorogenic acid, quercetin, and ferulic acid were the main free phenolic compounds. Furthermore, a positive correlation (r > 0.70) was observed between the identified peel phenolics and antioxidant activity (measured by DPPH, ABTS^+^, and FRAP assays), demonstrating once again the importance of phenolics as natural antioxidant compounds. This positive correlation between phenolic content and the antioxidant capacity was also reported in *H. undatus* peels [9].

Different extraction methods for the release of free phenolics bound to the peel of *H. undatus* were also employed by another study [7]. There was better bioactivity of the bound phenolic compounds when compared to the free phenolic compounds. Highlighting that the combination of alkaline extraction and ultrasound treatment increases the yield of polyphenols. The highlight was mainly attributed to the presence of daphnetin belonging to the coumarin class and derivatives. In the bound polyphenol extract, daphnetin was 120 times more abundant than in the free polyphenol extract. This compound exerts biological activities such as antioxidant, anti-inflammatory, antiarthritic effects, inhibition of pseudoallergic reactions, and improves cognitive function in mice, with potential application in the treatment of Alzheimer’s disease [29,30,31,34]. The authors also highlight the greater presence of medicarpin (a compound similar to isoflavonoids), especially in the extract of bound polyphenols. Medicarpine is associated with the prevention of postmenopausal arthritis [35], inhibits osteoclastogenesis [36], and has neuroprotective effects against Alzheimer’s disease [32].

Among the 57 selected studies, pitayas of the genus Hylocereus can be considered the most characterized fruits in terms of the presence of phenolic compounds. The species *Hylocereus polyrhizus* has been analyzed for the phenolic chemical profile by other studies [10,34,35]. In the peel and pulp of the species *H. polyrhizus*, up to 15 phenolic compounds have been identified, of which we can mainly mention the flavonoid class [34]. Flavonoids such as epicatechin, phlorizin, and kaempferol were identified in the fruit pulp. The marked presence of phenylpropanoids and flavonoids in *Hylocereus polyrhizus* pitaya is noteworthy in other studies [29,32,33,35,36]. Caffeic acid, along with quercetin and its derivatives, were compounds identified by most of the studies cited involving the species *H. polyrhizus*.

Pitayas’ fruits have numerous tiny, granular black seeds, which are usually eaten along with the pulp, which can be sources of phenolic compounds with antioxidant effects. In the species *H. polyrhizus*, a variety of phenylpropanoids have been identified, such as caffeic acid, ferulic acid, caffeic acid hexoside, synapic acid, and E-p-coumatic acid [29,36].

The phenolic composition of pitaya *Hyocereus megalanthus* (white pulp and yellow peel) was characterized by Ferreres et al. [15] and Paśko et al. [16]. In the first study, flavonoids were identified in the fruit peel. Notably, derivatives of quercetin and kaempferol. Both studies characterized the fruits as a potential source of phenolics [15]. In the pulp, the flavonoids myricetin, rutoside, and quercetin were identified [15].

The species *Hylocereus costaricensis* was also evaluated for the presence of phenolic compounds [16,33]. Flavonoids such as myricetin, rutoside, quercetin, pyrocatechol, rutin, and hispidulin were identified in the peel and pulp of the fruits. The great diversity of phenylpropanoids was also presented for this fruit. Compounds belong mainly to the class of benzoic acid derivatives and derivatives and hydroxycinnamic acids and derivatives. Sen et al. [33] concluded that the pulp and peel of *H. costaricensis* and *H. undatus* are rich sources of secondary metabolites, as well as food coloring compounds that can be exploited by various industries to replace synthetic bioactive agents.

The species *Selenicereus setaceus* Rizz., popularly known as “Saborosa do Cerrado”, had its pulp characterized as the presence of polyphenols [37]. In this fruit, large varieties of substances were found, such as hydroxycinnamic acids (chlorogenic acid, caffeic acid, ferulic acid, coumaric acid, and transcinnamic acid); flavonoids such as catechin, quercetin, and rutin were also identified. No studies were found on the identification of polyphenols in the *S. setaceus* peel.

**Table 1:** Profile of phenolic compounds identified in pitaya fruits.

Vegetable Fraction	Fruit	Technique	Extraction Method	Individual Compounds Identified	Reference
Pulp	*Stenocereus* spp.	HPLC-DAD ESI/MS	The extraction of the compounds was conducted using the solution of methanol:TFA (trifluoroacetic acid) 1% in water (80:20, *v*/*v*), followed by the ultrasonic bath for 30 min and left to rest at −20 °C for 3 h.	**(Flavonoids)** Quercetin 3-O-rutinoside, Kaempferol hexoside, Isorhamnetin hexoside, Isorahamnetin 3-O-glucoside Eriodictyol hexoside, Eriodictyol acetylhexoside, and Naringenin acetylhexoside	[22]
				**(Phenylpropanoids)** Caffeoyl hexoside I, Caffeoyl hexoside II, Feruloyl dihexoside, and *p*-coumaroyl quinic acids	
Pulp (seedless)	*Hylocereus undatus*	LC-ESI-MS/MS	Extraction with methanol by ultrasonic maceration, in three cycles of 30 min.	**(Flavonoids)** Rutin, hesperidin, kaempferol, isoquercitrin, and pinocembrin	[7]
				**(Phenylpropanoids)** Chlorogenic, Ferulic, Synapic, Caffeic, and Gallic acid	
Pulp (seedless)	*Hylocereus polyrhizus*	LC-ESI-MS/MS	Extraction with methanol by ultrasonic maceration, in three cycles of 30 min.	**(Flavonoids)** Rutin, Hesperidin, Kaempferol, and Pinocembrin	[7]
				**(Phenylpropanoids)** Chlorogenic, Ferulic, Synapic, Caffeic, and Gallic acids	
Peel	*Hylocereus undatus*	UPLC-QTOF-MS/MS	Extractions followed with 80% methanol, alkaline hydrolysis with NaOH solution at 60 °C + ultrasonic bath, finally, acid hydrolysis using ethyl acetate to obtain free and bound polyphenols.	**(Benzoic acid derivatives)** 7-[3,5-Dihydroxy-4-(4-hydroxyphenyl)-2-methoxyphenyl]-3-methoxy-3,4-dihydrooxepine-2,5-dione, Orsellinic acid, Homogentisic acid, Phenylpyruvic acid, 4-Vinylphenol and Drofenine, and 3,4-Dihydroxybenzoic acid	[9]
				**(Coumarins and derivatives)** Daphnetin	
				**(Phenylpropanoids)** 3-(4-Hydroxy-3-methoxyphenyl)-2-propenoic acid, Sinapoyl malate, Sinapic acid, Sinapic acid, and 3-(4-Hydroxyphenyl)-2-propenoic acids	
				**(Phenalenes)** 4,6,7-Trihydroxy-5-methoxy-1,8,8,9-tetramethyl-8,9-dihydro-3H-phenaleno[1,2-b]furan-3-one	
				**(Phenol esters)** 4-Hydroxyphenyl acetate **(Tannins)** [3,4,5-Trihydroxy-6-(hydroxymethyl)oxan-2-yl] 3-hydroxy-4,5-dimethoxybenzoate	
				**(Flavonoids)** Eupatilin	
				**(Hydroxy acids and derivatives)** 3-Hydroxysebacic acid **(Other)** Medicarpin	
Pulp seedless and peel	*Hylocereus polyrhizus*	UPLC-QTOF-MS^E^	Extraction with hexane, performed in an ultrasonic bath with a fixed power of 135 W for 20 min. The polar compounds were extracted in an ethanol/water solution (70:30, *v*/*v*).	**(Flavonoids)** Quercetin and Quercetin-3-O-hexoside	[34]
Peel	*Hylocereus undatus*	HPLC-DAD-ESI/MS^n^	Aqueous extraction was performed in a high-performance microwave digestion unit at 600 W. Different combinations of solid/solvent ratio, temperature, and extraction time were tested to optimize the extraction of phenolic compounds.	**(Flavonoids)** Kaempferol, Quercetin and Isorhamnetin derivatives **(Phenylpropanoids)** Cinnamoyl derivatives, *p*-coumaroyl derivatives, Caffeoylquinic acid derivatives, and Cinnamoyl quinic acids derivatives	[15]
Peel	*Hyocereus megalanthus*	HPLC-DAD-ESI/MS^n^	Aqueous extraction was performed in a high-performance microwave digestion unit at 600 W. Different combinations of solid/solvent ratio, temperature, and extraction time were tested to optimize the extraction of phenolic compounds.	**(Flavonoids)** Kaempferol, Quercetin, and Isorhamnetin derivatives **(Phenylpropanoids)** Cinnamoyl derivatives, *p*-coumaroyl derivatives, Caffeoylquinic acid derivatives, and Cinnamoyl quinic acids derivatives	[15]
Pulp	*Hylocereus undatus* and *Hylocereus polyrhizus*	UPLC-MS/MS	Extraction with aqueous ethanol (ethanol:water 80:20, *v*/*v*) using an ultrasonic bath for 30 min at 60 °C	**(Phenylpropanoids)** Caffeic, Ferulic, Protocatechuic, 2,4-di-OH benzoic, Vanillic, *p*-Coumaric, Sinapic, Gallic, *t*-Cinnamic, Salycylic, *o*-Coumaric, 3-OH benzoic, Ellagic, *p*-OH benzoic, Chlorogenic, and Syringic acids	[10]
Pulp	*Hylocereus costaricensis*, *H. undatus* and *H. megalanthus*	HPLC	The compounds were extracted with methanol or water for 3 h (stirring at 19 °C).	**(Flavonoids)** Miricetin, Rutoside, and Quercetin	[16]
				**(Phenylpropanoids)** Gallic, Cafeic and Protocatechuic acids	
Peel	*Hylocereus* spp.	LC-MS/MS	The peel compounds were extracted with a semi-continuous hydrothermal process of high pressure at 15 MPa at 60 °C at a water flow rate of 2 mL/min at pH 2.	**(Phenylpropanoids)** *p*-coumaric, Protocatechuic, and Piperonylic acids	[38]
Pulp	*Hylocereus polyrhizus*	UPLC-ESI-QTOF-MS^E^	The pulp was treated with Pectinex^®^ Ultra AFP (2000 mg L^−1^, treated at 4 °C, during 45 min at 150 rpm), and filtered through a microfiltration system	**(Flavanoides)** Quercetin-3-*O*-hexoside, Quercetin, and Luteolin	[35]
Pulp	*Hylocereus undatus*	HPLC	Methanol extraction.	**(Phenylpropanoids)** Protocatechuico, Cafeic, Clorogenic, and Galic acids	[39]
				**(Flavonoids)** Fhloridzin, Kaempferol, Rutin, and Epicatechin	
Pulp	*Hylocereus polyrhizus*	HPLC-DAD	The pulp was centrifuged (4000× *g* 15 min) and the supernatant was filtered through a 0.45 μm Millex-HA filter.	**(Phenylpropanoids)** Cafeic, Caftaric, Galic and Syringic acids	[28]
				**(Flavonoids)** Catechin, Epicatechin, Epigallocatechin gallate, Procyanidin B1, Procyanidin B2, Hesperidin Kaempferol 3-glucoside, Quercetin 3-glucoside, and Rutin **(Anthocyanins)** Cyanidin 3,5-diglucoside and Delphinidin 3-glucoside	
Pulp	*Hylocereus undatus*	HPLC-PDA	Extraction with methanol at 4 °C for 12 h.	(**Phenylpropanoids)** Galic, Protocatechuric, *p*-hydroxybenzoic, Caffeic and *p*-Coumaric acid	[40]
Pulp	*Hylocereus undatus*	HPLC-PDA	Extraction with methanol at 4 °C for 12 h.	**(Phenylpropanoids)** *p*-Coumaric, *p*-Hydroxybenzoic, Protocatechuric, Gallic, and Caffeic Acids	[41]
Pulp	*Hylocereus lemairei*	HPLC	Water extraction under the reflux system coupled to a condenser.	**(Flavonoids)** Quercetin, kaempferol	[30]
Peel	*Hylocereus lemairei*	HPLC	Water extraction under the reflux system coupled to a condenser.	**(Flavonoids)** Epicatechin, Quercetin, and Kaempferol	[30]
Pulp	*Hylocereus undatus*	HPLC	Extraction with methanol for 24 h at 4 °C.	**(Phenylpropanoids)** Protocatechuic, Chlorogenic, and Gallic acids	[39]
				**(Flavonoids)** Phloridin, Kaempferol, Rutin, and Epicatechin	
Seed	*Hylocereus polyrhizus*	UPLC-ESI-Q-TOF-MS^E^	Extraction with hexane + ultrasonic bath with a fixed power of 135 W for 20 min to obtain non-polar compounds. The polar compounds were extracted in an ethanol/water solution (70:30) under the same conditions as in the previous procedure.	**(Phenylpropanoids)** Caffeic, Ferulic, and Caffeic acid Hexoside	[36]
				**(Flavonoids)** Quercetin hexoside	
Pulp	*Hylocereus polyrhizus*	UPLC-ESI-Q-TOF-MS^E^	Extraction with hexane + ultrasonic bath with a fixed power of 135 W for 20 min to obtain non-polar compounds. The polar compounds were extracted in an ethanol/water solution (70:30) under the same conditions as in the previous procedure.	**(Flavonoids)** Quercetin hexoside	[36]
Pulp	*Hylocereus undatus*	HPLC	Extraction with methanol.	**(Phenylpropanoids)** Chlorogenic; Caffeic and Protocatechuic acids	[42]
				**(Flavonoids)** Epicatechin, Rutin, Phlorizin, and Kaempferol	
Pulp	*Hylocereus polyrhizus*	LC–MS/MS	Extraction with 50% ethanol.	**(Benzoic acid derivatives)** Ellagic acid	[32]
				**(Flavanoid)** Luteolin	
Pulp	*Hylocereus polyrhizus*	HPLC-DAD	Extraction with methanol: formic acid: water (50:5:45 *v*/*v*/*v*). The mixture was vortexed until smooth, then soaked at 4 °C for 15 min.	**(Flavonoids)** Miricetin and Rutin	[43]
				**(Phenylpropanoids)** Gallic acid	
Pulp	*Stenocereus huastecorum*	UPLC-MS/MS	Extraction with methanol (50%) assisted by an ultrasonic bath with ice for 4 min.	**(Phenylpropanoids)** Balsamic acid	[44]
Pulp	*Hylocereus polyrhizus*	HPLC-DAD/UV-Vis	Extraction with 70% aqueous methanol (*v*/*v*) followed by ultrasound sonication for 1 h at room temperature.	**(Phenylpropanoids)** Gallic, Catechin, Caffeic, and Vanillin acids	[45]
Peel and pulp	*Hylocereus costaricencis* and *Hylocereus udantus*	UHPLC	Extraction with 80% methanol, followed by heating in a boiling water bath for 2 h.	**(Benzoic acid derivatives)** Gallic, Syringic, Vanillic, Protocatechuic, and Ellagic acids	[33]
				**(Flavonoids)** Pyrocatechol, Rutin, Quercetin, and Hispidulin	
				**(Phenylpropanoids)** 3,4-dihydroxy cinnamic, Sinapic, caffeic, Ferulic, trans-cinnamic, and Chlorogenic acids	
Peel	*Hylocereus undatus*	HPLC	Extraction with methanol.	**(Phenylpropanoids)** *p*-Hydroxybenzoic, Ellagic Chlorogenic acids	[46]
				**(Flavonoids)** Catechin	
Pulp	*Hylocereus undatus*	HPLC	Extraction with methanol	**(Phenylpropanoids)** Protocatechuic, Gallic Caffeic, and Chlorogenic acids **(Flavonoids)** Phloridzin, Kaempferol, Rutin, and Epicatechin	[47]
Peel	*Hylocereus undatus*	HPLC–DAD	The powdered peel was mixed with either water or 60% ethanol and kept overnight at room temperature (27 °C) with constant stirring (400 rpm).	**(Phenylpropanoids)** Caffeic acid and Ferulic acid	[48]
				**(Flavonoids)** Quercertin	
Peel	*Hylocereus polyrhizus*	UPLC-QTOF/MS	The powdered peels were mixed with 82% ethanol and refluxed for 103 min at 56 °C.	**(Phenylpropanoids)** Gallic acid, Sinapic acid, and Rutin **(Flavonoids)** Quercetina-3-O-(6″-O-acetil)-*β*-D-glucopiranosídeo, Kaempferol-3-O -*β*-D-glucopiranosídeo, Malvidin-3-O-(6-O-acetil-*β*-D-glucopiranosídeo)-5-O-*β*-D-glucopiranosídeo and Isorhamnetina-3-O-(2G-α-L-ramnosil)-rutinosídeo	[49]
Pulp and peel	*Hylocereus undatus*	HPLC	The samples were mixed with methanol/water solution (80:20 *v*/*v*) and submitted to an ultrasonic bath at different times (5, 15, and 30 min), both under atmospheric conditions and under vacuum (12 mbar).	**(Phenylpropanoids)** Caffeic, *p*-Coumaric, Ferulic,	[50]
				Gallic, 3,4-Dihydroxybenzoic, and Syringic acids	
				**(Flavonoids)** Catechin, Rutin, and Quercetin	
				**(Stilbene)** Resveratrol	
Pulp	*Selenicereus setaceus* Rizz.	HPLC	Extraction with 70% methanol in an ultrasonic bath for 60 min.	**(Flavonoids)** Catechin, Quercetin, and Rutin	[37]
				**(Phenylpropanoids)** Gallic, Chlorogenic, Caffeic, Coumaric, Ferulic, and Trans-cinnamic acids	
Pulp	*Hylocereus polyrhizus* and *Hylocereus undatus*	HPLC	Samples were boiled for 1 h. After cooling, they were filtered and mixed with an acetone:water solution (3:1).	**(Flavonoids)** Quercetin, Kaempferol, and Myricetin	[51]
				**(Phenylpropanoids)** Caffeic acid, *p*-coumaric acid	
				Gallic acid, and Ellagic acid,	
Seeds	*Hylocereus polyrhizus*	UPLC-QTOF/MS	The seeds were dried, ground, and defatted with n-hexane. After hexane evaporation, the samples were extracted with ethanol, varying ethanol concentration, extraction time, and temperature.	**(Flavonoids)** Rutin, Kaempferol-3-O-rutinoside, Kaempferol-3-O-*β*-d-glucopyranoside, Apigenin-7-O-*α*-l-rhamnose (1→4)-6″-O-acetyl-*β*-d-glucoside, and Isorhamnetin-3-O-(2G-*α*-l-rhamnosyl)-rutinoside	[29]
				**(Phenylpropanoids)** Sinapic acid and *E*-*p*-Coumatic acids	
Pulp and peel	*Hylocereus undatus*	LC-MS	Ethanol extraction (95%) in Soxhlet	**(Flavonoids)** Rutin, Quercetin, and Kaempferol	[52]
				**(Phenylpropanoids)** 3,4-Dihydroxybenzoic,	
				Chlorogenic, Caffeic, Coumaric, and Ferulic acids	
Pulp	*Hylocereus polyrhizus*	UPLC-ESI-MS	The minimally processed fruits were ground into a powder with liquid nitrogen, and the extraction and centrifugation were repeated three times at 4 °C.	**(Flavonoids)** 3,4-Dihydroxybenzaldehyde, Catechin, Epicatechin, Protocatechuic acid, Nicotiflorin, Rutin, Narcissin, Hesperidin, Naringenin, and Naringin	[53]
				**(Phenylpropanoids)** *trans*-Cinnamic, Vanillic, 4-Hydroxybenzoic, Salicin, Salicylic,	
				4-Hydroxycinnamic, Caffeic, Chlorogenic, Cryptochlorogenic, Ferulic, and Sinapic acids	
Pulp (seedless)	*Stenocereus thurberi*	UPLC-DAD-MS	The pulp was macerated in distilled water, then sonicated for 27 min and stirred for 20 min.	**(Phenylpropanoids)** Ferulic, Caffeic, p-Coumaric, Caffeoylquinic, and	[20]
				Gallic acids	
				**(Flavonoids)** Catechin, Rutin, Isorhamnetin, Quercetin, and Glycosylated quercetin	
Peel	*Hylocereus undatus*	HPLC	The pulp puree was extracted with methanol under constant stirring (200 rpm) at 25 °C/24 h.	**(Phenylpropanoids)** *p*-hydroxybenzoic acid Ferulic acid	[54]

The flavonoids commonly observed in pitaya fruits, such as isorahmetin, quercetin, and kaempferol, are the main active components of Chinese medicines for the treatment of COVID-19 [55,56].

**Figure 8:**
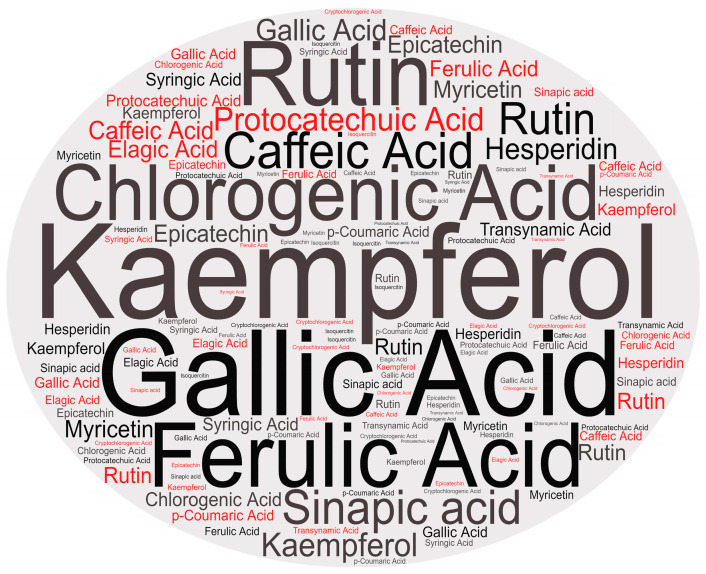
Word cloud with the main phenolic compounds identified in the studies. Generated by: https://makewordcloud.com/.

The most commonly identified compounds in pitaya fruits are described in Figure 8. Phenylpropanoids were the most listed compound in most studies, followed by the flavonoid class. These results show that the fruits of these cacti have a heterogeneous chemical profile, with compounds that can help in beneficial effects on health.

The main compounds identified in pitayas have beneficial effects on health associated with their bioactivities. These studies show that, in general, cactus fruits can be good sources of phytochemicals of a phenolic nature. Several prominent flavonoids identified in pitaya, such as kaempferol and rutin (Table 2, images 1–14), possess a range of health-promotion properties. Kaempferol exhibits antihypertensive, cardioprotective, antioxidant, and anti-inflammatory effects [57]. Additionally, it has been shown to protect against microvascular diseases induced by high blood sugar levels [58] and may play a role in reducing alcohol-induced liver damage [59]. Rutin has also been reported by many studies. Research suggests that this compound alleviated ventilator-induced lung injury [60] and may offer protection against kidney injury by improving oxidative stress and regulating lipid metabolism [61].

Pitaya’s phenolic acid profile contributes significantly to its potential health benefits. Gallic acid can be a natural inhibitor of α-amylase, making it a promising candidate for the development of diabetic-friendly functional foods and nutraceuticals [62]. They demonstrate neuroprotective effects [63] and therapeutic properties in obesity management [64].

Ferulic acid (Table 2, Image 22) offers a range of health benefits. It can improve glucose metabolism, protect the gut barrier by regulating the gut microbiota [65], and exert antioxidant, hepatoprotective, and antihyperglycemic effects [66]. Chlorogenic acid, another key phenolic acid in pitaya, possesses antioxidant and anti-inflammatory properties and improves lipid metabolism [67,68].

Caffeic acid (Table 2, Image 23) stands out for its potential applications in treating various conditions. It has been shown effectiveness in managing cancer [69], diabetes [70], and Parkinson’s disease [70]. Protocatechuic acid (Table 2, Image 31), another phenolic acid identified in pitaya, demonstrates antidiabetic activities [71] and potential chemopreventive properties [72].

Pitaya fruits boast another unique group of compounds, betalains, responsible for the captivating colors of their pulp and peel. Beyond their aesthetic appeal, betalains hold promise for human health, as suggested by various studies [34,73,74]. Furthermore, their natural origin has generated significant interest in their potential application as food colorants [35,75].

In recognition of the multifaceted importance of these compounds, most studies characterize their content in detail. The following section delves into the betalain profiles identified in various pitaya fruits.

### 2.2. Profile of Betalains

Betalains belong to the class of alkaloids derived from tyrosine, restricted to certain fungi, and an order of vascular plants [76]. They are natural water-soluble pigments, present in some plant species, such as beets (Beta vulgaris) and those of the *Cactaceae* family of the genera *Epiphyllum*, *Hylocereus*, *Opuntia* [77], and *Stenocereus* [22,78]. Beyond their vibrant colors, betalains contribute significantly to the health potential of pitaya fruits. These phytochemicals exert several beneficial effects on health due to their anticancer, anti-inflammatory, antilipidemic, antimicrobial, antioxidant, and anxiolytic properties [34,73,77,79]. More than 50 betalain compounds have been described in the literature [22]. Some of these are presented in Table 3, present in the different species of pitayas characterized in the selected studies.

A total of 16 species of pitaya were reported (Table 3), belonging to the genera *Hylocereus* sp., *Stenocereus* sp., and *Opuntia* sp., with *Hylocereus polyrhizus* (red pulp and pink peel) being the most characterized in terms of the presence of betalains, followed by *H. undatus* (white pulp and pink peel) [77].

All studies included in this analysis identified and/or quantified betacyanins, the major subclass of betalains, while betaxanthins were identified in only 11 studies (Table 3). Furthermore, betacyanins exhibited greater structural diversity within the pitaya betalain profile. Betanins and their derivatives were consistently detected in both the peel and pulp.

Barkociová et al. [77] identified betanin, isobetanin, and phyllocactin as the principal betalains in a survey of 19 pitayas samples representing varius species. Similar findings were reported for *H. undatus* peels [15] and both peel and pulp fractions of *H. polyrhizus* [12], where betanin and phyllocactin dominated the betalain profile. Roriz et al. [18] quantified four betacyanins in *H. costaricensis* peel and observed the highest amount of phyllocactin (14.8 mg/g extract), followed by isobetanin (11.8 mg/g), isophylloctin (11.7 mg/g), and betanin (10.1 mg/g). Bethanin was also the betacyanin with the highest concentration in the mature pulp and peel of *H. costaricensis* and in the peel of *H. undatus* [33].

Some authors, such as García-Cruz et al. [22], quantified betalains and determined the levels of total betacyanins and betaxanthins by HPLC-DAD-MS in the *Stenocereus pruinosus* orange (162.07 and 22,053.46 μg/g) and red (5423.38 and 17,706.72 μg/g) pulps and in the *S. stellatus* red pulp (2525.82 and 21,681.66 μg/g), respectively. In the lyophilized pulp of *H. polyrhizus*, Cheok et al. [43] found 139.19 mg/100 g (dry basis) of total betalains and 108.14 mg/100 g (dry basis) of betacyanins, using a combination of spectrophotometric (UV-visible at λ = 538 nm) and chromatographic (HPLC-DAD) methods.

Song et al. [81] employed spectrophotometry at a wavelength of 538 nm to quantify betacyanins in a purified powdered extract of *H. undatus* peel, obtaining a yield of 25.32 mg/g. Sen and Baruah [33] found the following concentrations of total betacyanins: 28.6 mg/100 g (on a wet basis) in mature pulp, 21.9 mg/100 g in *H. costaricensis* peel, and 10.5 mg/100 g in *H. undatus* peel by spectrophotometry at λ = 536 nm.

The consumption of fresh or industrial processing of pitayas generates about 35% of waste, composed mostly of the peel of the fruit. These commonly discarded biowastes are promising sources of bioactive compounds, such as betalains [18]. Thus, some authors suggest its use as a potential source of betalains [12,33,80]. Suh et al. [80] found that the *Hylocereus polyrhizus* and *Hylocereus undatus* peels had higher amounts of betacyanins and betaxanthins than the pulp. As observed by Younis et al. [73], all samples of H. undulatus peel extracts, from different origins, exhibited higher concentrations of betacyanins than the pulp extracts. On the other hand, Sen and Baruah [33] found that the mature pulp of *H. costaricensis* contained more total betacyanins, betanin, phyllocatin, betadine-5-O-hydroxybuteryl glucoside, and isohylocerrin than the peel.

The preceding discussion highlights the significant variation in betalain content observed across pitaya species, cultivars, geographical origins, and even between fruit parts. This emphasizes the importance of identifying and quantifying betalains in each fruit analyzed within a research study.

### 2.3. Health Effects

The modern lifestyle, characterized by significant changes in dietary habits, has contributed to a dramatic rise in chronic non-communicable [86]. Diets high in processed, refined foods with excessive sugar and fat, coupled with inadequate vegetable intake, are key factors associated with this growth [87]. Fruits and vegetables are essential components of a healthy diet, and their regular consumption in sufficient amounts is linked to the prevention of several diseases, including diabetes, obesity, cardiovascular diseases, and some types of cancer [88,89].

Emerging research suggests that pitaya fruits hold promise due to their potential functional activities and various bioproperties. As summarized in Table 4, these include cytotoxic potential (anti-tumor effects) [16], antinociceptive and antiproliferative of cancer cells [13], antioxidant [44], and anxiolytic [34].

Several studies link the presence of various bioactive compounds, including phenylpropanoids, flavonoids, and betalains [91,92]. Pasko et al. [16] aimed to qualitatively and quantitatively evaluate the compounds with bioactive properties present in different species of pitayas (*Hylocereus costaricensis*, *Hylocereus undatus*, and *Hylocereus megalanthus*). For this, the antioxidant capacities of plant materials were determined, as well as the potential cytotoxic activity to several normal and cancer cell lines, grouped as: skin panel, prostate panel, gastrointestinal panel, along with lung carcinoma. The authors reported that the fruits showed high antioxidant property and selective cytotoxic activity, mainly related to cancer cells of the gastrointestinal tract [16]. Thus, as reported by Ramírez-Rodrigues et al. [44], they also verified antioxidant and anti-apoptotic effects in their study. Further supporting the antiproliferative effects of red pitaya extracts against cancer cells, Marques et al. [13] observed a potent to moderate effect on sarcoma 180 (S-180) cells, suggesting potential biopharmaceutical applications. In a separate study, Salam et al. investigated the apoptotic (cell death-inducing) potential of *H. undatus* peel and pulp extracts in MCF-7 and Caco-2 cancer cell lines. Their findings revealed selective cytotoxic activity against breast and colorectal cancer cells, and the authors linked this activity to the presence of phenolic compounds and flavonoids in the extracts [52].

According to the World Health Organization (WHO), in recent years the global prevalence of anxiety and depression has increased by 25%, being one of the most common mental disorders in the world, with a prevalence of 3.6% of the population [93]. Considering the importance of these disorders, scientific studies have been carried out with the aim of evaluating the anxiolytic activity of different substances, including those found in pitaya fruits [34].

Lira et al. [34] suggest that H. polyrizhus has anxiolytic phytotherapeutic potential in adult zebrafish, being a plant-derived alternative. In addition, they report that pitaya peels, usually discarded during industrial processing, can be considered a considerable product with high economic added value aimed at combating anxiety disorders.

The pitaya peel accounts for about 33% of the total weight of the fruit. Although it is a raw source of betalains, studies focused on its chemical composition and nutritional quality are still limited [81]. However, there are those scientific studies that show the bioactive potential of these plant materials, and report their potential beneficial effect on health through the ability to improve obesity, insulin resistance, and steatosis, exemplified in the work of Song et al. [81], by the inhibitory effect of α-amylase and α-glucosidase, as described by Zhong et al. [9], for the high antioxidant capacity with photoprotection property, enunciated by Vijayakumar et al. [49] and for the therapeutic effects in an oxidative-inflammatory microenvironment that mimics diabetes combined with metformin [30].

It is also possible to report other in vivo studies that potentiate the beneficial effects of extracts obtained from pitayas. According to Holanda et al. [36], treatment with pitaya by mice resulted in an increase in HDL cholesterol and a decrease in total and LDL cholesterol, triacylglycerols, glycemia, alanine aminotransferase, and aspartate aminotransferase, related to the beneficial effect on the cholesterol and dyslipidemic profile of these animals. Additionally, Macias-Ceja et al. [32] demonstrated that the ethanolic extract of *H. polyrhizus* exerts anti-inflammatory activity and prevents trinitrobenzenesulfonic-induced colitis in mice, suggesting that it may be a therapeutic alternative for inflammatory diseases.

According to Cheok et al. [43], cardiovascular diseases are currently the leading cause of mortality and a ubiquitous cause of morbidity in many countries. In view of the above, these authors aimed to investigate, for the first time, the effects of acute and short-term consumption of dietary amounts of pitaya (powdered fruit) on vascular function in healthy men and women, elucidating the cardioprotective potential of betalains.

A double-blind, randomized, placebo-controlled, crossover trial was conducted with nineteen healthy volunteers (8 males and 11 females) who were non-smokers, aged between 18 and 40 years, and observed that the consumption of lyophilized pitaya, in nutritionally achievable amounts, may have the potential to clinically improve endothelial function and arterial stiffness. This effect can be attributed to the high content of betalain present in the fruit [43].

### 2.4. Application in Food Matrices

The fruits, in addition to being consumed in natura, can be used as ingredients in the preparation of new food products, as well as their by-products, such as peels and seeds. The use of fruits in food matrices, in addition to increasing their shelf life and adding value, seeks to help improve sensory perceptions, such as flavor, odor, and color, generally enriching the product nutritionally and sensorially [94]. In addition, the use of peels is in line with the concept of making full use of food, minimizing losses and waste [95].

Regarding pitayas, some studies have addressed their application as a natural dye [18,35,75] in the preparation of juices [44,82,96] and as an ingredient in bread [54]. These studies are summarized in Table 5.

Pitaya fruits can be consumed, mainly in natura, but also as minimally processed products and juices, due to seasonality and perishability [82], as well as a functional natural additive for the food industry [75].

Increasingly, the food industry is looking for natural food dyes to replace synthetic ones, as these can pose a risk to the health of the population [75]. This is because the color of a food is attractive to the consumer, which can influence their purchasing power, in addition to being related to sensory quality, potential biological activities, and health benefits [82]. There are several substances from plants that perform this function of giving color to products, such as anthocyanins, carotenoids, chlorophylls, and betalains [35].

The different species of *Hylocereus* spp. differ mainly by the color of the pulp and peel. As an example, we can mention *H. constaricensis*, which has red pulp and reddish-pink pulp; *H. megalanthus*, with white flesh and yellow peel; *H. undatus*, with white pulp and pink peel [18]; and *H. polyrhizus*, with purple-red flesh and rind [35]. The colors of these fruits are related to the presence of betalains: betaxanthins (yellow color) and betacyanins (purplish color), which favors the use of pulp and peel in the development of natural food colors [18,35].

In this context, Gengatharan, Dykes, and Choo [75] and Lima et al. [35] evaluated the application of dyes obtained from pitaya pulp in yogurts. These authors used different methods to obtain these dyes. In the study of Gengatharan, Dykes, and Choo [75], pitaya red pulp was extracted with distilled water and pectinase for 2 h and, after filtration and centrifugation, the supernatant was dehydrated at 40 °C for 24 h. Then, the dye obtained was added to cow’s milk, and, after pasteurization and cooling, the starter culture (*Streptococcus thermophilus* and *Lactobacillus delbrueckii* ssp. bulgaricus) was inoculated and incubated at 37 °C for 6 h. The yogurt obtained contained 125 g/kg of betacyanin.

The microfiltration process was used to obtain the pitaya dye in the study by Lima et al. [35]. The red pitaya pulp was treated with pectinase at 4 °C for 45 min at 150 rpm and subjected to microfiltration (pore diameter of 0.2 μm). The microfiltrate was concentrated under vacuum until it reached 63 °Brix, and the dye obtained was added to commercial natural yogurt in different concentrations (0.5 to 2%). The yogurt formulations with coloring had a similar sensory profile and were accepted by the panelists.

Betacyanins are very unstable during processing and storage [82]. Thus, Liao et al. [82] proposed the use of thermosonication in the pitaya juice production process to maintain the original purple-red color. These authors varied the temperature (10 to 70 °C) and time (5 to 40 min) and found that the thermosonication preserved the color of the juice better than the industrial heat treatment (83 °C/1.5 min) and the control (50 °C/20 min).

Another interesting application reported in the literature was pitaya juice added to probiotic strains of *Lactobacillus rhamnosus*, developed by Usaga et al. [96]. The commercial pitaya pulp was thawed, filtered, heated to 93 °C for 0.1 min and hot-bottled, with the subsequent addition of *L. rhamnosus*. The minimum count (>106 CFU/mL) of these strains remained viable for 19 days at 5 °C, as well as the levels of betalains. Therefore, the use of pitaya is a promising alternative for the plant-based functional food market [96].

The incorporation of fermented pitaya into breads has been studied by Omedi et al. [54]. The pitaya pulp was homogenized into a puree, where the Pediococcus pentosaceus culture was inoculated and fermented at 30 °C for 24 h. Subsequently, this yeast was incorporated in different concentrations (5, 15, and 25%) into bread dough containing mung beans, wheat flour, and other ingredients. As a result, breads with fermented pitaya had a higher content of soluble dietary fiber, carbohydrates, and total flavonoids. In the in vivo study, the higher concentrations of fermented pitaya contributed to a healthier gut microbiota in the mice. According to these authors, this prebiotic effect is due to the polyphenols and soluble dietary fiber present in the matrix of mung beans and fermented pitaya. In addition, in the animals fed the formulation with 15% fermented pitaya, an improvement in glucose intolerance and an increase in the HDL: LDL profile were observed, in addition to anti-inflammatory effects.

In view of the above, the versatility of the application of pitaya can be verified, especially in the development of natural additives and functional foods. In addition, the consumption of pitaya both in natura and incorporated in different formulations contributes to the beneficial effects on the consumer’s health.

## 3. Materials and Methods

The methodology for the elaboration of the integrative review was based on the previous study by Ramos et al. [97] and da Veiga Correia [87]. Initially, searches were carried out in four databases: ScienceDirect, PubMed, Scielo, and Periódicos CAPES. Only indexed and research articles published in the last ten years, available in Portuguese, English, or Spanish, were included in the study. The selection of articles was carried out in October and November 2023 by three researchers.

The third stage was conducted with the pre-selection of articles, based on the established search strategies (Table 6). The fourth stage was based on the inclusion and exclusion criteria, applied after reading the title and abstract, where information that answered the guiding question had been described without the need to read the article in full. Finally, in the fifth stage, after reading the articles in full, only the selected articles that could answer the guiding question and had the potential to assist in conducting the study were interpreted and discussed.

## 4. Conclusions

The different genera and species of pitayas show a high content of health-promoting phytochemicals and desirable technological applications. Among the most characterized species in terms of polyphenol content are *Hylocereus undatus* and *Hylocereus polyrhizus*. The variety of phenolic chemicals found in the pulp characterizes pitayas as “super fruits”, with great bioactivity and an alternative source of extraction of natural pigments for application in the food industry, such as betalains. Fruit peels also present a potential source for the extraction of bioactives, making them a valuable biowaste and a viable alternative for the use of a wide variety of functional chemicals.

The main phenolic compounds identified in these fruits are flavonoids and a wide variety of phenylpropanoids from the classes of hydroxycinnamic and benzoic acid derivatives and derivatives. Kaempferol and rutin were the most reported flavonoids, as well as phenolic, chlorogenic, caffeic, protocatechuic, synaptic, and ellagic acids.

This work may guide other researchers regarding the phytochemical composition of cactus fruits and their residual fractions, as well as better ways of extracting and quantifying the compounds and their pharmaceutical and food industry applications.

## Figures and Tables

**Table 2:** Basic structures of some classes of phenolic compounds found in pitayas.

**Flavonoids**		
**1** 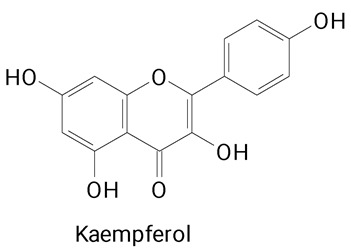	**2** 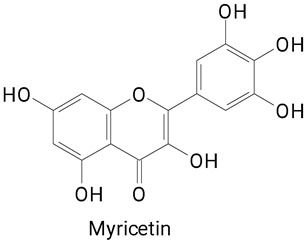	**3** 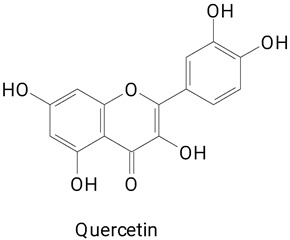
**4** 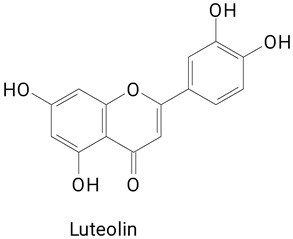	**5** 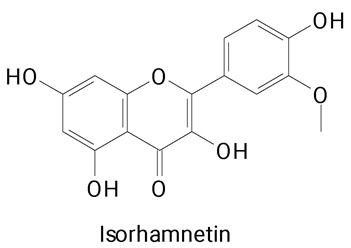	**6** 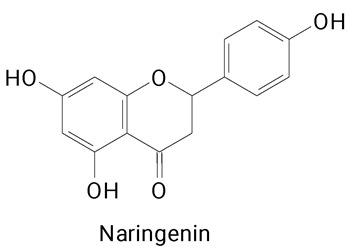
**7** 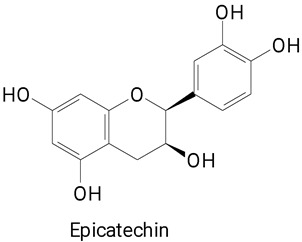	**8** 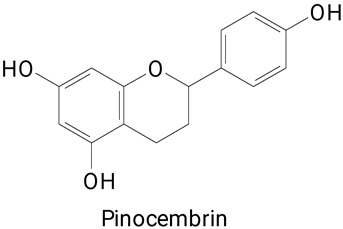	**9** 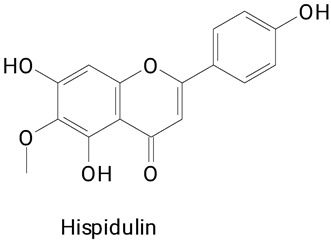
**10** 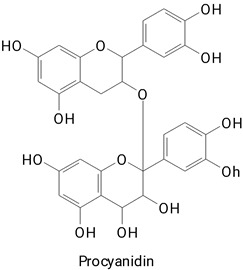	**11** 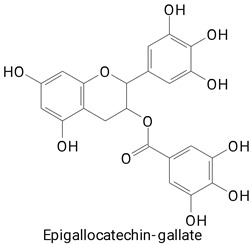	**12** 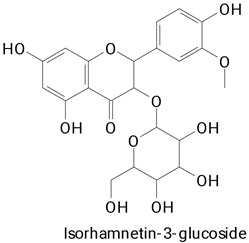
**13** 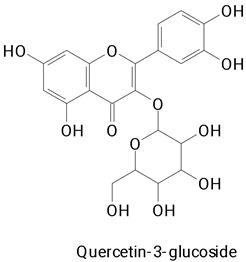	**14** 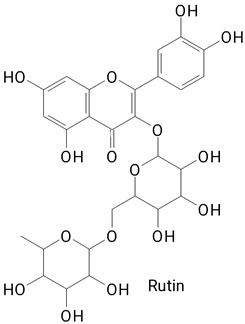	**15** 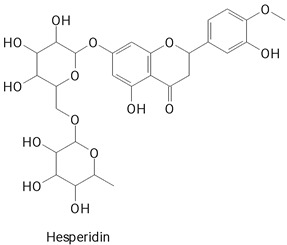
**16** 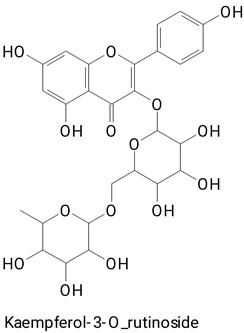	**17** 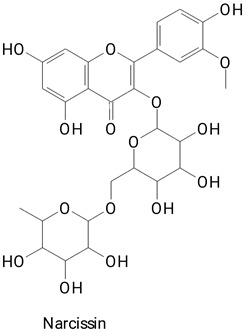	**18** 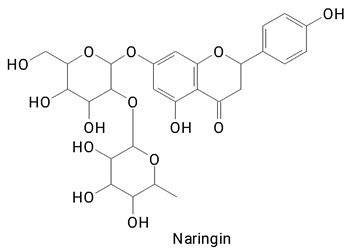
**19** 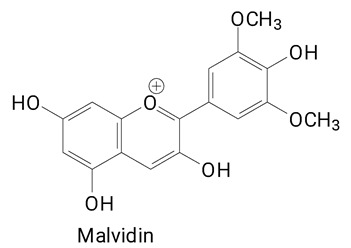		
**Phenylpropanoids**		
**20** 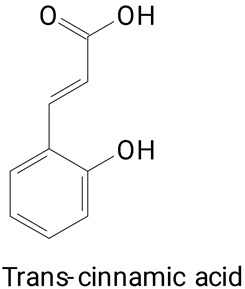	**21** 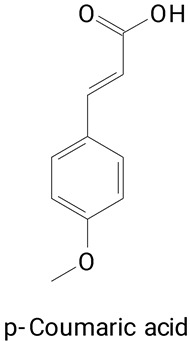	**22** 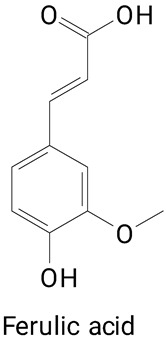
**23** 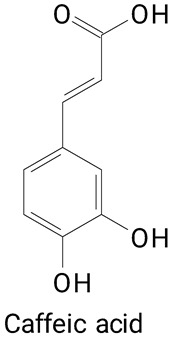	**24** 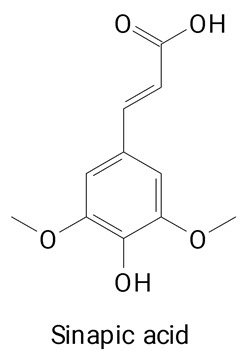	**25** 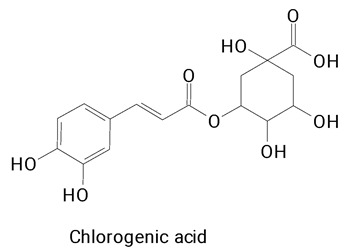
**26** 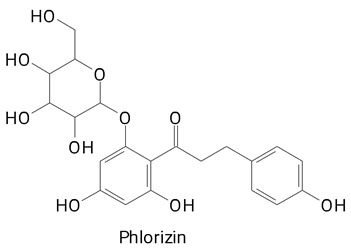	**27** 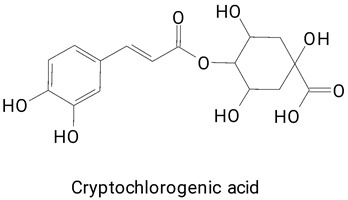	**28** 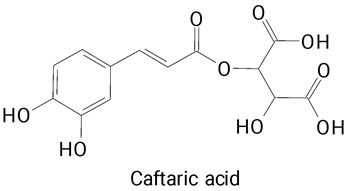
**29** 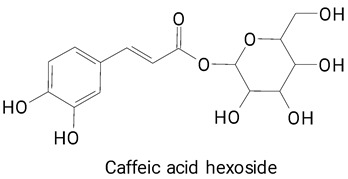		
**Benzoic acid derivatives**		
**30** 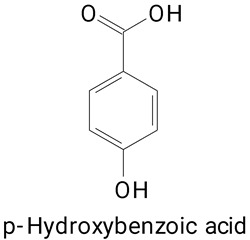	**31** 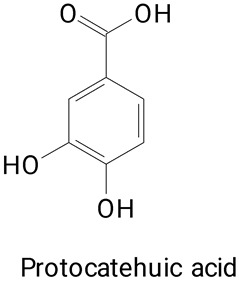	**32** 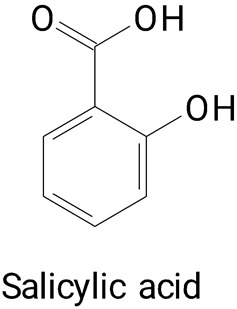
**33** 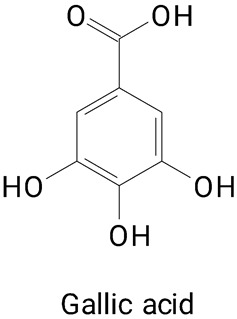	**34** 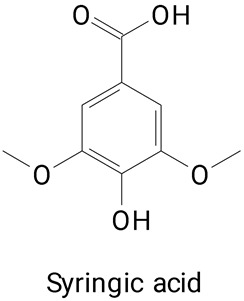	**35** 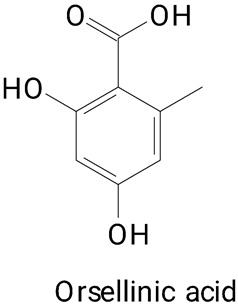
**36** 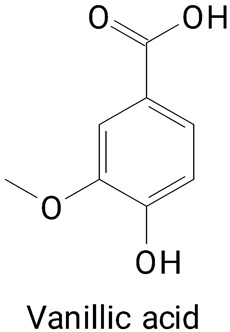	**37** 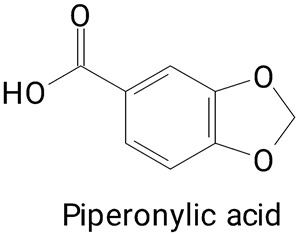	**38** 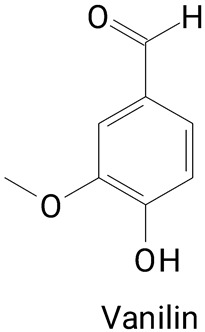
**39** 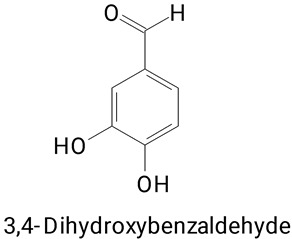	**40** 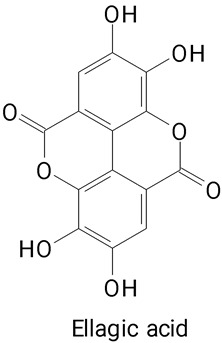	
**Coumarins**		
**41** 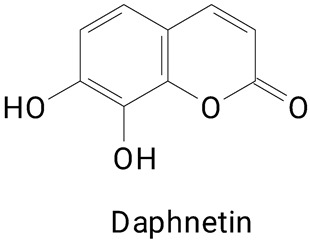		**42** 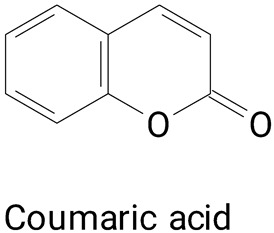
**Betalains**		
**43** 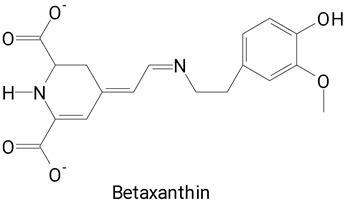	**44** 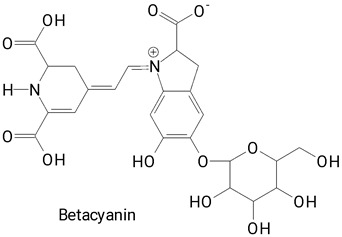	
**45** 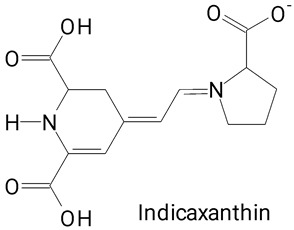	**46** 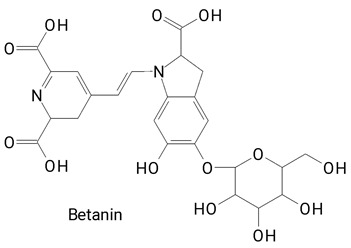	
**Others**		
**47** 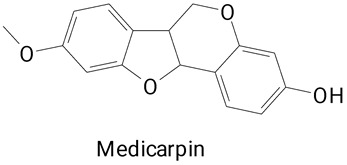	**48** 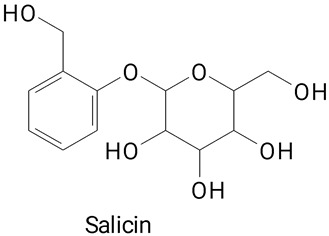	**49** 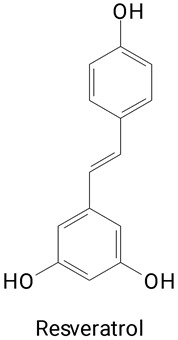

**Table 3:** Profile of betalains compounds identified in pitaya fruits.

Species and Fraction	Technique	Betalains	Reference
*Hylocereus polyrhizus* and *Hylocereus undatus*. (Peel and pulp)	UPLC−Q-TOF−MS and LC−ESI−MS/MS	**(Betacyanins)** Betanin, Decarboxylated betanin, Phyllocactin, Decarboxylated phyllocactin, Neobetanin, Decarboxylated neobetanin, Decarboxylated hylocerenin, Neophyllocactin, Betanidin, Tridecarboxylated neobetanin, Lampranthin II, Tridecarboxylated hylocerenin, Decarboxylated neohylocerenin, Dehydrogenated, Tridecarboxylated neobetanin, Dehydrogenated, Decarboxylated neohylocerenin, Neohylocerenin, and Bidecarboxylated hylocerenin	[80]
		**(Betaxanthins)** Indicaxanthin, Dopaxanthin, Miraxanthin V, 3-Methoxytyramine−betaxanthin, Phenylalanine−betaxanthin, and Portulacaxanthin II	
*Hylocereus polyrhizus* (Peel)	UHPLC–ESI/MS/MS	**(Betacyanins)** Betanidine 5-O-b-sophoside, Betanin, Isobetanin, Apiosyl-betanin, Phyllocactin (6′-O-malonylbetanine), Butyrylbetanin, Hylocerenin (3-hydroxy-3-methyl-glutaryl-betanin), Isophyllocactin, Iso-butyryl betaine’ 2′-apiosyl-phyllocactin, and Isohylocerenin’ 2′-apiosyl-isophyllocatin.	[12]
(Pulp)		Betanidine 5-O-b-sophoside, Betanin, Isobetanin, apiosyl-betanin, Phyllocactin’(6′-O-malonylbetanine), Butyrylbetanine, Hylocerenin(3-hydroxy-3-methyl-glutaryl-betanine), Isophylolactiniso-butyryl betaine, and Isohylocerenin	
*Hylocereus undatus* (Peel)	HPLC−ESI/MS/MS	**(Betacyanins)** Betanin, Isobetanin, 17-decarboxy-betanin, 17-decarboxy-isobetanin, Phyllocactin, Betanidin-5-O-(6′-O-3-hydroxy-butyryl)-*β*-glucoside, 2-decarboxy-betanin, 17-decarboxy-phyllocactin, Isophyllocactin, 2′-O-apiosyl-phyllocactin, 17-decarboxy-isophyllocactin, 2-decarboxy-phyllocactin, and 2-decarboxy-isophyllocactin	[81]
*Hylocereus undatus* and *H. megalanthus*. (Peel)	HPLC-DAD-ESI/MS	**(Betacyanins)** Betanin, Isobetanin, Phyllocactin, and isophyllocactin	[15]
*Stenocereus pruinosus* and *S.* *stellatus* (Pulp)	HPLC-DAD ESI/MS	**(Betacyanins)** Gomfrenin I, Isogophrenine I, 2-decarboxy-betanin, Phyllocactin, 4-O-malonyl-betanine or Betanidine-5-O-(6′-O-3-hydroxy-butyril)-*β*-glucoside, Isophyllocactin, 60-O-malonyl-2-descarboxybetanin, Betanidin derivative, and 6-O-malonyl-2-decarboxy-isobetanine	[22]
		**(Betaxanthins)** Isoindicaxanthin and Indicaxanthin	
*Hylocereus polyrhizus* (Pulp)	LC/MS/MS	**(Betacyanins)** Betanin, Isobetanin, Betanidin, Phyllocactin, and Hylocerenin	[75]
*Hylocereus polyrhizus* (Pulp)	HPLC	**(Betacyanins)** Betanin and Isobetanin	[79]
*Hylocereus polyrhizus* (Pulp)	UPLC-DAD-ESI/MS	**(Betacyanins)** Betanin, Isobetanin, 2′-O-apiosil-betanina, Filocactina, Betanidin-’-O-(6′-O-hidroxibutiril)-*β*-glicosídeo, Isobetanidin-’-O-(6′-O-hidroxibutiril)-*β*-glicosídeo and Isofilocat’na, and 2′-O-apiosil-filocactina	[82]
*Hylocereus polyrhizus* (Pulp)	UPLC-ESI-QTOF-MS	**(Betacyanins)** Phyllocactin, Apiosyl-malonyl-betanin, Betanin, and apiosyl-malonyl-betanine	[35]
*Hylocereus polyrhizus* (Peel and pulp)	UPLC-QTOF-MS	**(Betacyanins)** Phyllocactin, Isophyllocactin, Betanin, Isobetanin, 2′-O-Apiosyl-phyllocactin, and 6′-Malonyl-2′-descarboxy-betanin	[34]
*Hylocereus polyrhizus*, *H. megalanthus* e *H. undatus* (Peel and pulp)	UHPLC-MS/MS	**(Betacyanins)** Betanin	[8]
*Hylocereus costaricensis*	LC-MS/MS	**(Betacyanins)** Betanin, Isobetanin, 2′-apiosyl-betanin, 2′-apiosyl-isobetanin, Phyllocactin, 4′-O-malonyl-betanin, Isophyllocactin, 2′-O-apiosyl-phyllocactin, and 2′-O-apiosyl-isophyllocactin; **(Betaxanthins)** *γ*-aminobutyryl acid-betaxanthin and Indicaxanthin.	[77]
*Hylocereus undatus*		**(Betacyanins)** Betanin, Isobetanin, 2′-apiosyl-betanin, 2′-apiosyl-isobetanin, Phyllocactin, 4′-O-malonyl-betanin, Isophyllocactin, and 4′-O-malonyl-isobetanin; **(Betaxanthins)** Indicaxanthin	
*Hylocereus* sp.		**(Betacyanins)** Betanin, Isobetanin, 2′-apiosyl-betanin, 2′-apiosyl-isobetanin, Phyllocactin, 4′-O-malonyl-betanin, Isophyllocactin, Hylocerenin, 2′-O-apiosyl-phyllocactin, Isohylocerenin, and 2′-O-apiosyl-isophyllocactin **(Betaxanthins)** *γ*-aminobutyryl acid-betaxanthin and Indicaxanthin.	
*Opuntia aurea*		**(Betacyanins)** Betanidin 5-O-*β*-sophoroside, Betanin, Isobetanidin 5-O-*β*-sophoroside, Isobetanin, Gomphrenin I, Phyllocactin, and Isophyllocactin **(Betaxanthins)** Indicaxanthin.	
*Opuntia camanchica*		**(Betacyanins)** Betanin, Isobetanin, 2′-apiosyl-betanin, 2′-apiosyl-isobetanin, Gomphrenin I, Phyllocactin, 4′-O-malonyl-betanin, Isophyllocactin, Hylocerenin, 4′-O-malonyl-isobetanin, 2′-O-apiosyl-phyllocactin, and 2′-O-apiosyl-isophyllocactin; **(Betaxanthins)** Indicaxanthin.	
*Opuntia crinifera*		**(Betacyanins)** Betanidin 5-O-*β*-sophoroside, Betanin, Isobetanidin 5-O-*β*-sophoroside, Isobetanin, 2′-apiosyl-betanin, 2′-apiosyl-isobetanin, Phyllocactin, 4′-O-malonyl-betanin, Isogomphrenin I, 4′-O-malonyl-isobetanin, 6′-O-sinapoyl-glucosyl-betanin, and 6′-O-sinapoyl-glucosyl-isobetanin; **(Betaxanthins)** γ-aminobutyryl acid-betaxanthin and Indicaxanthin.	
*Opuntia fragilis*		**(Betacyanins)** Betanin, Isobetanin, Gomphrenin I and Phyllocactin	
*Opuntia humifusa*		**(Betacyanins)** Betanin, Isobetanin, Gomphrenin I, Phyllocactin, Isogomphrenin I, and Isophyllocactin; **(Betaxanthins)** *γ*-aminobutyryl acid-betaxanthin and Indicaxanthin.	
*Opuntia polyacantha*,		**(Betacyanins)** Betanidin 5-O-*β*-sophoroside, Betanin, Isobetanidin 5-O-*β*-sophoroside, Isobetanin, Gomphrenin I, Phyllocactin, Isogomphrenin I, and Isophyllocactin; **(Betaxanthins)** Indicaxanthin	
*Opuntia tomentella*		**(Betacyanins)** Betanin, Isobetanin, Gomphrenin I, Phyllocactin; *Betaxantinas: γ*-aminobutyryl acid-betaxanthin, and Indicaxanthin	
*Opuntia zacuapanensis* (Fresh fruits)		**(Betacyanins)** Betanin, Isobetanin, Gomphrenin I, Phyllocactin, and Isogomphrenin I **(Betaxanthins)** *γ*-aminobutyryl acid-betaxanthin and Indicaxanthin	
*Hylocereus polyrhizus* L. Peel	HPLC-PDA	**(Betacyanins)** Betanin, Isobetanin, phyllocactin, Isophyllocactin, Butyrylbetanin, Isobutyrylbetanin, 2′-apiosyl-phyllocactin, and 2′-Apiosyl-isophyllocactin	[83]
*Stenocereus pruinosus* Pulp	HPLC	Betacyanins and betaxanthins	[78]
*Hylocereus polyrhizus* Pulp	UPLC-ESI-Q-TOF-MS	**(Betacyanins)** Betanin, Phyllocactin, Isobetanin, Isophylloccatin, Apiosyl-malonyl-betanin, 6′-O-Malonyl-2-decarboxy-betanine isomer, and Phyllocactin derivative; **(Betaxantinas)** Indicaxanthin	[36]
Seeds		**(Betacyanins)** Phyllocactin, Isophylloccatin, 6′-O-Malonyl-2-Decarboxy-Betanin Isomer and Phyllocactin Derivative, and Apiosyl-Malonyl-Betanin	
*Hylocereus polyrhizus* (Pulp and peel)	LC-MS	**(Betacyanins)** Betanin, Isobetanin, Phyllocactin, 4′-Malonyl-betanin, Isophyllocactin, and Neobetanidin 5-O-(6′-O-3″-hydroxy-3″-methylglutaryl)-*β*-glucoside tri-decarboxylated;	[84]
		**(Betaxanthins)** Indicaxanthin	
*Hylocereus undatus*, *H. polyrhizus* (Pulp and peel)	UPLC-MS/MS	**(Betacyanins)** Gomphrenin I, Phyllocactin II, isophyllocactin, and Betanin	[85]
*Hylocereus polyrhizus* (Pulp)	HPLC-DAD	**(Betacyanins)** Betanin, Isobetanin	[43]
*Stenocereus huastecorum* (Pulp)	UPLC-MS/MS	**(Betacyanins)** Betanin, Phyllocactin and Isophyllocttin	[44]
		**(Betaxanthins)** Indicaxanthin	
*Hylocereus costaricensis* (Peel)	UPLC−ESI/MS/MS	**(Betacyanins)** Betanin, Isobetanin, Phyllocactin, and Isophyllocactin	[18]
*Hylocereus polyrhizus* (Peel, pulp and seeds)	UPLC-Q-TOF-MS	**(Betacyanins)** Betanin, Isobetanin, Phyllocactin, and Isophyllocactin	[13]
*Hylocereus costaricensis* (Peel and pulp)	UHPLC	**(Betacyanins)** Betanin, Isobetanin, Phyllocatin, Betadine-5-Ohydroxybuteryl glucoside, and Isohylocerrin	[33]
*Hylocereus undatus* (Peel)			
*Hylocereus undulatus* (Peel)	UPLC-ESI–qTOF-MS	**(Betacyanins)** Tridecarboxy-neohylocerenin, Decarboxy-neophyllocactin, Dehydrogenated tridecarboxy-neophyllocactin, Bidecarboxy-hylocerenin, Betanidin, Dehydrogenated tridecarboxy-neobetanin, Decarboxy-phyllocactin, Isobetanin, Bidecarboxy-neophyllocactin, Apiosyl phyllocactin, Apiosyl-betanin, Tridecarboxy-betanin, Dehydrogenated decarboxy-neobetanin, Decarboxy-neobetanin, Bidecarboxy-neohylocerenin, Apiosyl-isobetanin, Dehydrogenated tridecarboxy-neohylocerenin, Tridecarboxy-neobetanin, Bidecarboxy phyllocactin, Betanin, Neophyllocactin, Betanidin dihexoside, Dehydrogenated bidecarboxy-neohylocerenin, Neobetanin, Dehydrogenated decarboxy-neohylocerenin, Decarboxy-neohylocerenin, Prebetanin, Phyllocactin, Decarboxy-hylocerenin, Hylocerenin, Neohylocerenin, Dehydrogenated bidecarboxy-neophyllocactin, Tridecarboxy-phyllocactin, Decarboxy-isobetanin, Tridecarboxy-neophyllocactin, Bidecarboxy-betanin, Dehydrogenated decarboxy-neophyllocactin, Decarboxy-betanin, and Betanidin malonyl dihexoside	[73]
		**(Betaxanthins)** Portulacaxanthin II, Vulgaxanthin III, Indicaxanthin, Portulacaxanthin III, Muscaaurin VII, Vulgaxanthin II, Miraxanthin III, Vulgaxanthin I, Miraxanthin I, Miraxanthin II Methoxytyramine−betaxanthin, γ-Aminobutyric acid-betaxanthin, and Vulgaxanthin IV	
(Pulp)		**(Betacyanins)** Bidecarboxy-hylocerenin, Isobetanin, Bidecarboxy-neophyllocactin, Apiosyl phyllocactin, Tridecarboxy-hylocerenin, Apiosyl-betanin, Tridecarboxy-betanin, Decarboxy-neobetanin, Bidecarboxy-neohylocerenin, Dehydrogenated tridecarboxy-neohylocerenin, Tridecarboxy-neobetanin, Bidecarboxy phyllocactin, Betanin, Betanidin dihexoside, Dehydrogenated bidecarboxy-neohylocerenin, Neobetanin, Tridecarboxy-phyllocactin, Decarboxy-isobetanin, Tridecarboxy-neophyllocactin, Bidecarboxy-betanin, and Decarboxy-betanin	
		**(Betaxanthins)** Portulacaxanthin II; Vulgaxanthin III, Indicaxanthin, Portulacaxanthin III, Muscaaurin VII, Vulgaxanthin II, Miraxanthin III, Miraxanthin V, Miraxanthin I, Miraxanthin II Methoxytyramine−betaxanthin, and Vulgaxanthin IV	

**Table 4:** Bioactivity associated with the phytochemical content of pitayas.

Vegetable Fraction	Fruit	Biological Effects/Mechanism	Main Results	Reference
Pulp	*Hylocereus costaricensis*, *H. megalanthus*, and *H. undatus*	Cytotoxic activity for cancer cells (In vitro study)	Selective cytotoxic activity, directed primarily against cancer cells of the gastrointestinal tract.	[16]
Pulp and peel	*Hylocereus polyrizhus*	Anxiolytic effect (Animal model study)	*H. polyrizhus* has the potential as an alternative plant-derived anxiolytic therapy.	[34]
Peel, pulp and seeds	*Hylocereus polyrhizus*	Antinociceptive (Animal model study)	The seeds ethanolic extract displayed antinociceptive activity at the inflammatory phase, similar to morphine.	[13]
Pulp and seeds	*Hylocereus polyrhizus*	Beneficial effect on the cholesterolemic profile of dyslipidemic in rats (Animal model study)	Treatment with pitaya resulted in an increase in HDL cholesterol, and a decrease in total and LDL cholesterol, triacylglycerols, blood glucose, alanine aminotransferase, and aspartate aminotransferase.	[90]
Whole Fruit Powder	*Hylocereus polyrhizus*	Improved vascular function in men and women (Human study model)	The dragon fruit consumption, at nutritionally in achievable amounts, may have the potential to provide clinically meaningful improvement in endothelial function and arterial stiffness, possibly attributed to its high betalain content.	[43]
Pulp	*Stenocereus huastecorum*	Antioxidant and anti-apoptotic effects (Animal model study)	Treatment with concentrated pitaya juice (PJC) improves functional and structural damage in cisplatin-treated rats by altering the generation of reactive oxygen species (ROS) and activating the NO pathway.	[44]
Peel and pulp	*Hylocereus undatus*	Potential apoptotic activities in MCF-7 and Caco-2 cancer cell lines (In vitro study)	Both extracts can induce apoptosis in vitro.	[52]
Peel	*Hylocereus undatus*	Ability to improve obesity, insulin resistance, and steatosis (Animal model study)	Purified betacyanins from pitaya peel reduced body weight gain induced by a high-fat diet and improved adipose tissue hypertrophy, hepatosteatosis, glucose intolerance, and insulin resistance.	[81]
Pulp	*Hylocereus polyrhizus*	Anti-inflammatory effects and prevention of murine colitis (Animal model study)	Systemic administration of the ethanolic extract of *H. polyrhizus* exerts an anti-inflammatory effect and prevents murine colitis induced by 2,4,6-trinitrobenzenossulfônico (TNBS).	[32]
Peel	*Hylocereus undatus*	Inhibitory effect of α-amylase and α-glucosidase (In vitro study)	Bound polyphenol extracts showed a significant inhibitory effect on α-amylase, and α-glucosidase was stronger compared to free polyphenol extracts.	[9]
Peel	*Hylocereu lemairei*	Effect of pitaya on endothelial cells under elevated glucose levels, mimicking hyperglycemia. (In vitro study)	Pitaya peel extract can be used as a co-therapeutic strategy to minimize oxidative damage and inflammation in endothelial cells under elevated glucose levels.	[30]
Peel, pulp and seeds	*Hylocereus polyrhizus*	Effects of excessive and short-term consumption of *Hylocereus polyrhizus* fruit on vascular function in a healthy population. (Human study model)	Acute and short-term consumption of dragon fruit in viable amounts in the diet improved endothelial function and arterial stiffness in healthy individuals.	[43]
Peel	*Hylocereus polyrhizus*	Antioxidant and photoprotective effect (In vitro study)	*Hylocereus polyrhizus* peel extract is a potent antioxidant with excellent photoprotective properties. In addition, the phenolic and flavonoid compounds of HPPE contributed to the overall antioxidant activities, as well as to the high SPF value and broad-spectrum UVA and UVB photoprotection.	[49]

**Table 5:** Potential use of pitaya in the preparation and incorporation of food products.

Species and Fraction	Product Developed	Main Results	Reference
*Hylocereus polyrhizus* Pulp	Powdered food coloring added to yogurt	Yogurt with pitaya coloring showed betacyanin, antioxidant activity, and reduced syneresis. The sensory evaluation indicated acceptability of the yogurt color.	[75]
*Hylocereus polyrhizus* Pulp	Microfiltered food coloring added to yogurt	The dye contained mainly carbohydrates, flavonoids, and betalains and showed microbiological and physicochemical stability for 12 weeks. The sensory evaluation of the yogurt indicated an improvement in the quality of the color without affecting the aroma and other attributes.	[35]
*Hylocereus costaricensis* Peel	Dye powder	The high presence of betacyanins can be highlighted as a promising natural alternative to artificial food colorings because, in addition to providing a certain color, it also has antioxidant and antibacterial effects of relevance for food preservation.	[18]
*Hylocereus polyrhizus* Pulp	Juice	The juice obtained after the thermosonication process showed better color protection capacity. There was decomposition of betacyanins and formation of Maillard reaction products, which were related to color stability.	[82]
*Stenocereus huastecorum* Pulp	Juice concentrate	The concentrated juice showed bioactive compounds and antioxidant and antiapoptotic effects. In the in vivo study, renal toxicity decreased.	[44]
*Hylocereus* sp. Pulp	Added juice of probiotic strains	Pitaya juice with the probiotic strain of *Lactobacillus rhamnosus* showed no change in betalain concentration during 19 days of cold storage.	[96]
*Hylocereus undatus* Pulp	Mungu bean bread with fermented pitaya puree	The bread with fermented pitaya had a higher content of soluble dietary fiber and essential active compounds. In the in vivo study in mice, there was an increase in the production of short-chain fatty acids and the HDL:LDL profile, promoted anti-inflammatory properties, and improved glucose intolerance.	[54]

**Table 6:** Search strategies.

Database	Search Strategy	Number of Works
ScienceDirect	(Pitaya) OR (*Hylocereus*) AND (phenolics) (Pitaya) OR (Dragon Fruit) AND (phenolics) (*Hylocereus*) AND (chemical profile) (*Hylocereus*) AND (bioactive compounds) (Pitaya) AND (flavonoids)	356
PubMed	(Pitaya) AND (phenolics) (Pitaya) OR (*Hylocereus*) AND (phenolics) (Pitaya) OR (*Selenicereus*) (Dragon Fruit) AND (phenolics) (Pitaya) AND (bioactive compounds)	71
Scielo	(Pitaya) OR (*Hylocereus*) AND (phenolics) (*Hylocereus*) OR (Pitaya) (phenolics) AND (Dragon Fruit) (bioactive compounds) AND (pitaya) (phenolics) AND (*Selenicereus*) OR (*Hylocereus*)	46
Capes Periodics	(Pitaya) OR (*Hylocereus*) AND (phenolics) (Pitaya) OR (Dragon Fruit) (phenolics) OR (bioactive compounds) AND (Pitaya) (phenolics) AND (Dragon Fruit) (flavonoids) AND (Pitaya)	128

## Data Availability

Not applicable.
